# Documented Nicotine, Cannabis, and Co-Use Are Associated With More Intensive Emergency Department Care for Adolescent Asthma Exacerbations

**DOI:** 10.1016/j.acepjo.2026.100453

**Published:** 2026-06-29

**Authors:** Hayes Stancliff, Anxhelo Mara, Sreyansh Rishabh, Stephen Sandelich

**Affiliations:** 1 Department of Medical Education, Penn State College of Medicine, Hershey, Pennsylvania, USA; 2 Penn State Harrisburg, Middletown, Pennsylvania, USA; 3 Departments of Emergency Medicine and Pediatrics, Penn State Health Milton S. Hershey Medical Center, Hershey, Pennsylvania, USA

**Keywords:** asthma, adolescent, substance-related disorders, emergency service, hospital, respiratory insufficiency

## Abstract

**Objectives:**

Asthma exacerbations pose substantial risk to adolescents. We evaluated whether documented nicotine use, cannabis use, or concurrent use is associated with increased emergency department (ED) management intensity and adverse short-term outcomes during asthma exacerbations.

**Methods:**

Using the TriNetX database, we identified adolescents aged 11 to 21 years presenting to the ED with an acute asthma exacerbation from January 1, 2015, to January 1, 2025. Patients were categorized into nicotine-only, cannabis-only, or concurrent nicotine-cannabis cohorts based on documented International Classification of Diseases, 10th Revision (ICD-10) codes and matched to controls without such diagnoses. Outcomes were reported as risk ratios (RRs) with 95% CIs during 0 to 1-day and 1- to 30-day intervals.

**Results:**

After matching, cohorts included 4604 nicotine-only, 2679 cannabis-only, and 1045 co-use patients. During their ED encounter, co-use patients demonstrated the strongest associations with acute respiratory failure (RR, 4.63; 95% CI, 2.71-7.89), hospital admission (RR, 2.47; 95% CI, 1.80-3.38), and critical care services (RR, 1.92; 95% CI, 1.19-3.09). Nicotine-only and cannabis-only cohorts also showed increased pharmacologic escalation and diagnostic testing. Between 1 and 30 days, co-use patients had higher rates of repeat exacerbation (RR, 1.72; 95% CI, 1.36-2.17), ED revisits (RR, 1.54; 95% CI, 1.29-1.85), pneumonia (RR, 2.20; 95% CI, 1.05-4.62), and critical care use (RR, 2.07; 95% CI, 1.10-3.90); nicotine-only and cannabis-only cohorts additionally demonstrated this association to a lesser degree.

**Conclusion:**

Adolescents with documented nicotine or cannabis use, especially concurrent use, experience more intensive ED management and worse short-term outcomes during asthma exacerbations, supporting consideration of targeted acute care pathways.

**The Bottom Line**

This study asked whether documented nicotine use, cannabis use, or both together are linked to worse outcomes during asthma attacks in adolescents seen in the emergency department. Using a large national database, we found that adolescents with documented substance use had more severe asthma-related complications and required more intensive hospital care compared with those without such histories. These patients were more likely to need respiratory support, hospital admission, and repeat emergency visits within 30 days. Overall, documented substance use was associated with higher-risk asthma exacerbations and worse short-term outcomes in this population.

## 1 Introduction

### 1.1 Background

The current prevalence of asthma globally is approximately 10% in both children and adolescents, making it the most common chronic disease in children globally.^1^ Within the US, asthma afflicts millions of children and is a large public health burden.^2^ It has been well-established that adolescents with asthma exhibit higher mortality rates and utilize health care resources more frequently.^3^ In addition to acute exacerbations and hospitalizations, asthma has deleterious effects on children’s quality of life, school attendance, and long-term respiratory health.^4^ Evidence suggests that adolescents with severe asthma experience higher rates of psychological distress, mental health disorders, and engagement in risk-taking behaviors.^5^

Among adolescents in the US, nicotine and cannabis use is a growing public health concern. Most recent estimates indicate that approximately 18% of middle and high school students report cannabis use, and 8.1% indicated current use of tobacco products.^6^^,^^7^ Co-use of tobacco and cannabis is additionally a frequent concern in this population and is associated with higher rates of other substance use and risk behaviors.^8^^,^^9^ The evolving landscape of substance use, including the rise of e-cigarette and cannabis vaping products, represents a potential source of long-term health-related consequences for adolescent populations.^7^

### 1.2 Importance

The interconnected nature of inhalant substance use and asthma outcomes among adolescents is increasingly recognized as clinically important, with most recent evidence elucidating a dose-response relationship between higher frequency of cannabis use and higher prevalence of asthma.^10^ Large-scale meta-analyses and systematic reviews have shown associations between cannabis consumption and respiratory symptoms such as wheezing, dyspnea, cough, and sputum production.^11^ In regards to tobacco, it has been well established that both active smoking and environmental tobacco smoke exposure are associated with poorer asthma control, exacerbations, and hospitalizations among adolescent populations.^12^^,^^13^ E-cigarette use has also been linked to increased risk of asthma symptoms, diagnosis, and exacerbations.^14^ Despite this growing body of evidence, the specific contribution of nicotine and cannabis use to acute asthma exacerbation management and short-term outcomes in emergency department (ED) settings remains incompletely characterized in large pediatric cohorts.

### 1.3 Goals of this Investigation

This study aimed to evaluate whether documented use of nicotine and/or cannabis through International Classification of Diseases, 10th Revision (ICD-10) codes was associated with differences in ED care management, health care utilization, and short-term clinical outcomes among adolescents presenting with asthma exacerbations. We hypothesized that patients with nicotine-only, cannabis-only, or concurrent nicotine-cannabis diagnoses would experience greater treatment intensity, higher rates of hospital admission and critical care use, and increased asthma-related re-presentation within 30 days compared with matched controls without such diagnoses.

## 2 Methods

### 2.1 Study Design

This retrospective cohort study used a propensity score-matched design using data from the de-identified TriNetX Research Network. TriNetX is a federated platform that aggregates real-time clinical information from participating health care organizations (HCOs). The network used for this analysis comprises data from 111 HCOs across the US and is specifically structured to support real-world evidence research.^15^ To maintain anonymity, the network does not permit identification of individual patients or institutions, while still allowing access to precise patient counts. Participating HCOs must meet TriNetX’s inclusion criteria: they must actively treat patients, use electronic health record (HER) systems, and engage in research or clinical trials. These organizations provide standardized data on diagnoses, procedures, medications, laboratory values, and demographic characteristics, encoded using ICD-10 codes, Logical Observation Identifiers, Names, and Codes (LOINC), RxNorm, and Current Procedural Terminology (CPT) terminologies. Data were accessed by the study investigators through the TriNetX analytics platform using standardized queries of deidentified EHR data. The TriNetX network aggregates data directly from participating health care organizations, where diagnoses, procedures, and medications are recorded as part of routine clinical care and billing processes. As such, data are not manually abstracted for research purposes but are derived from real-world clinical documentation. Because data are deidentified and queried at the aggregate level, investigators do not have access to individual patient charts, and traditional blinding of data abstractors is not applicable. This study manuscript follows the Strengthening the Reporting of Observational Studies in Epidemiology (STROBE) guidelines for retrospective cohort studies.^16^

### 2.2 Study Participants

This study analyzed male and female patients aged 11 to 21 years who presented to the ED (CPT code, 1013711) between January 1, 2015, and January 1, 2025, with a diagnosis of acute asthma exacerbation (ICD-10 code, J45.901 or J45.902). The age range was selected based on the American Academy of Pediatrics’ definition of adolescence, which encompasses individuals aged 11 to 21 years.^17^ The interval of 2015-2025 was chosen to maximize sample size and capture national trends in adolescent substance use; however, it is important to recognize that this interval encompasses temporal changes in nicotine use patterns and increasing cannabis legalization. Substance use exposures were therefore defined using standardized diagnostic codes available within the TriNetX network, and analyses reflect documented diagnoses rather than specific modes of use (eg, combustible vs vaping).

Patients were categorized into substance-specific exposure cohorts based on ICD-10 documented codes recorded within the preceding year. Three mutually exclusive exposure groups were defined, and included those with: (1) documented nicotine-related disorder (ICD-10: F17) without documented cannabis-related codes (“nicotine-only”); (2) documented cannabis-related disorder (ICD-10 code, F12) without documented nicotine-related codes (“cannabis-only”); and (3) concurrently documented nicotine and cannabis-related disorders (“co-use”). Each exposure cohort was compared with a corresponding control group of patients with asthma exacerbation who had no documented nicotine- or cannabis-related diagnostic codes in their medical records during the preceding year. ICD-10 diagnostic codes for nicotine- and cannabis-related disorders reflect clinically documented diagnoses rather than all substance use.

### 2.3 Outcome Measurements

Short-term outcomes of interest included hospital admission and the need for critical care services from the ED, acute respiratory failure, systemic corticosteroid use, inhaled beta-agonist use, magnesium sulfate administration, continuous positive airway pressure (CPAP) initiation, chest x-ray, blood gas analysis, and sedative use. The operational definitions for grouped terms were informed by existing literature and established clinical conventions. Through the use of such codes, we attempted to capture patients undergoing intensive management of their asthma exacerbation, defined by a composite of markers reflecting higher-acuity emergency care, including acute respiratory failure, hospital admission, use of critical care services, pharmacologic escalation, and increased diagnostic testing. This operational definition was selected based on existing Global Initiative for Asthma (GINA) guidelines that specify these markers as indications for advanced clinical care.^18^ The systemic corticosteroids isolated in this study included prednisone, dexamethasone, and prednisolone, which represent the most likely pharmacologic medications within this class to be used in the setting of an asthma exacerbation.^16^ The administration of an inhaled beta-agonist was defined as the use of albuterol or levalbuterol, which represent the 2 most commonly used medications in this class.^12^ We defined pharmacologic sedation as the administration of medications commonly used in the ED to manage acute agitation, facilitate procedures, or support care in patients with severe illness. This grouping included ketamine; benzodiazepines (eg, midazolam, lorazepam, and diazepam); and antipsychotic or sedating agents (eg, haloperidol, droperidol, olanzapine, ziprasidone, and promethazine). These medications are routinely used in ED practice for rapid sedation in scenarios such as severe distress or agitation interfering with care or to enable time-sensitive interventions, including airway management. Given the limitations of the database, medication indication cannot be directly ascertained; therefore, this variable was conceptualized as a marker of clinical severity or need for escalation of care rather than a condition-specific therapeutic intervention.^19^ Documented social determinants of health (SDOH) are captured in ICD-10 Clinical Modification (ICD-10-CM) codes Z55-Z65, encompassing problems related to education and literacy (Z55); employment and unemployment (Z56); occupational exposure (Z57); housing and economic circumstances (Z59); social environment (Z60); upbringing (Z62); primary support group, including family circumstances (Z63); psychosocial circumstances (Z64); and social exclusion and rejection (Z65). Assignment of Z-code SDOH diagnoses was used to capture documented SDOH within the EHR; however, given variability in institutional coding practices and known underutilization of these codes, this measure reflects recognition and documentation of social risk rather than a direct measure of underlying social need. Despite their limitations, SDOH codes have been used in existing ED literature to help identify patients who may require more intensive social support. A complete list of outcomes and their corresponding operational definitions is provided in Table S1.

Intermediate outcomes of interest included re-presentation to the ED, repeat asthma exacerbation, critical care services, acute respiratory failure, social determinant of health code assignment, and pneumonia within 1 day to 1 month of presentation.

### 2.4 Statistical Analysis

Statistical analyses were performed using the built-in analytic functions available within the TriNetX platform. For each outcome of interest, comparative risk analyses were conducted to assess the proportion of patients experiencing the outcome in each cohort. Results were reported as risk estimates with corresponding risk ratios (RRs) and 95% CIs. When appropriate, patients with a documented history of the outcome before the index event were excluded to focus on new (incident) cases following the index encounter. Given the exploratory nature of the study and the evaluation of multiple outcomes across cohorts and time intervals, no formal adjustment for multiple comparisons was applied. Results should therefore be interpreted in the context of potential type I eRR, or inflation. To minimize confounding, propensity score matching (PSM) was applied. Matching was conducted in a 1:1 ratio using logistic regression-derived propensity scores based on baseline demographic and clinical variables, including age, race, ethnicity, and relevant comorbidities.

**Figure 1:**
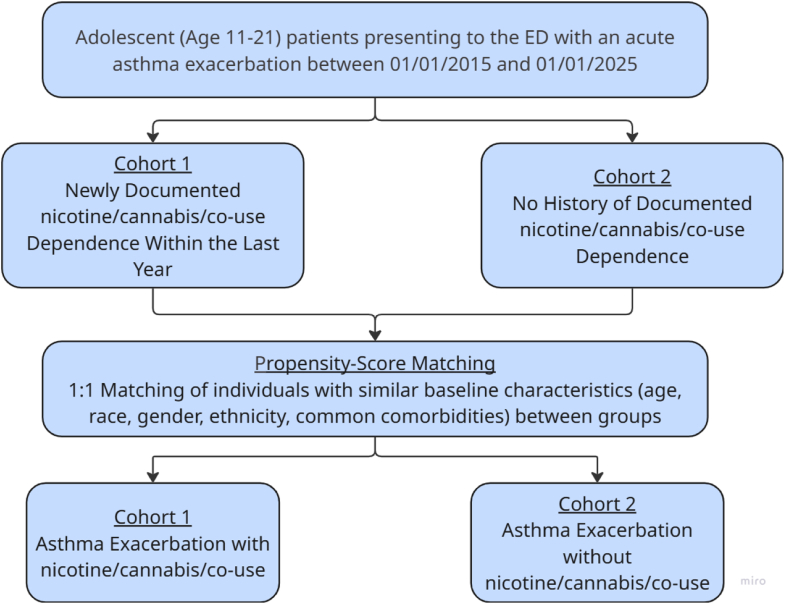
Flowchart of TriNetX query design. ED, emergency department.

We used 2 separate methods of determining which comorbidities were relevant to our population and could potentially influence outcomes. The first method was by controlling specifically for factors that contribute to worse outcomes in asthma exacerbations as established by existing literature. These included age, race, gender, ethnicity, and comorbidities.^6^ We additionally wanted to capture generally comorbid conditions prevalent in childhood that could influence these outcomes. These PSM variables were sourced from the Elixhauser Comorbidity Index, a validated ICD-10 coding system for capturing comorbidities developed by the Agency for Healthcare Research and Quality.^20^ These included sickle cell disorders (D57); congenital malformations of the circulatory system (Q20-Q28); coagulation defects and other hemoRR,hagic conditions (D65-D69); mood disorders (F30-F39); anxiety and related nonpsychotic disorders (F40-F48); behavioral and emotional disorders with onset in childhood and adolescence (F90-F98); developmental disorders (F80-F89); overweight and obesity (E65-E68); chromosomal abnormalities (Q90-Q99); congenital musculoskeletal malformations (Q65-Q79); metabolic disorders (E70-E88); cardiomyopathy (I42); conduction disorders (I45); chronic intestinal diseases (K55-K64); chronic lower respiratory diseases (J40-J4A); neoplasms (C00-D49); and diseases of the genitourinary system (N00-N99). Study cohort design is represented in Figure 1 below.

### 2.5 Ethical Considerations

The manuscript was exempt from review, and a waiver has been granted by the Penn State Institutional Review Board (STUDY00028040). The need for consent to participate was waived by the same Institutional Review Board. This retrospective analysis used deidentified data and was conducted in compliance with institutional policies and the ethical standards set forth in the Declaration of Helsinki.

## 3 Results

### 3.1 Baseline Patient Characteristics

#### 3.1.1 Documented nicotine-only cohort

Before PSM, the nicotine-only cohort included 4607 patients compared with 69,126 controls. After matching, 4604 patients remained in each group. Prior to matching, patients with documented nicotine use were older, with a higher proportion aged 15 to 21 years and fewer aged 10 to 15 years (all *P* < .001), along with modest differences in sex and race/ethnicity. Substantial prematching imbalances were observed in psychiatric comorbidities (mood, anxiety, and childhood behavioral disorders) and in genitourinary disease, metabolic disorders, and obesity, whereas several congenital and developmental conditions were more common in controls. After matching, demographic and clinical characteristics were well balanced, with standardized mean differences generally below accepted thresholds. Of note, the age variable remained statistically significantly different between exposure and control groups after matching in all 3 cohorts (eg, nicotine, 19.3 ± 2.21 vs 18.1 ± 2.55; *P* < .001). While statistically significant differences in mean age persisted between exposure and control groups, the absolute differences were small (approximately 1-1.2 years across cohorts), and the standardized mean differences for age remained below conventional thresholds for meaningful imbalance. This result is represented graphically in Table S2.

#### 3.1.2 Documented cannabis-only cohort

The patients with documented cannabis use comprised 2681 patients before matching and 2679 after matching, compared with 71,052 controls. Prior to matching, cannabis-exposed patients were older and more frequently aged 15 to 21 years (all *P* < .001), with small differences in sex and race/ethnicity distributions. Marked prematching imbalances were present in psychiatric comorbidities, genitourinary disease, metabolic disorders, obesity, behavioral disorders of childhood, and chronic lower respiratory disease. Following matching, demographic characteristic distributions and comorbidities were closely aligned across groups. This result is represented graphically in Table S3.

#### 3.1.3 Documented co-use cohort

Before matching, the nicotine-cannabis co-use cohort included 1048 patients and 1045 after matching, compared with 66,336 controls. Co-use patients were substantially older before matching, with a much higher proportion aged 15 to 21 years and fewer aged 10 to 15 years (all *P* < .001), while sex and race/ethnicity differences were modest. The largest prematching imbalances involved psychiatric conditions, genitourinary disease, metabolic disorders, obesity, and chronic lower respiratory disease, with additional differences in select hematologic and cardiac diagnoses; several congenital and developmental conditions were more common in controls. After propensity score matching, balance across demographic and clinical characteristics improved substantially, with standardized mean differences reduced to minimal levels. This outcome is represented graphically in Table S4.

### 3.2 Outcomes From 0 Day to 1 Day Postindex Visit

**Figure 2:**
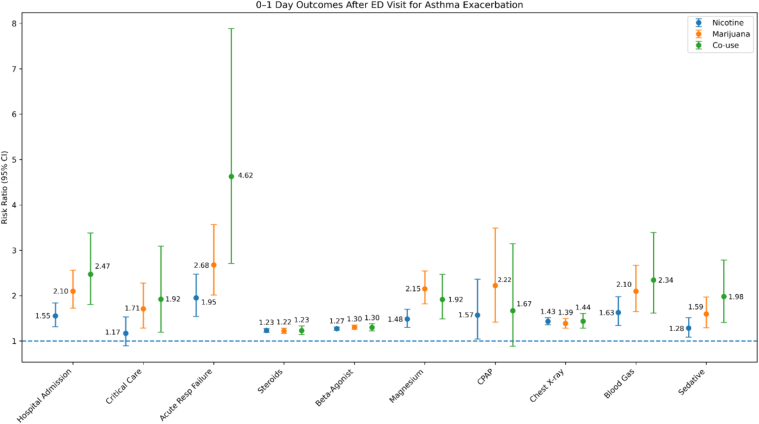
The 0- to 1-day outcomes after emergency department (ED) presentation for asthma exacerbation. Forest plot of risk ratios (RRs) with 95% CIs. Numeric RRs are displayed next to the upper CI bars; the dashed line indicates RR = 1.0.

During the first day following the index ED visit, all 3 substance-specific cohorts demonstrated higher rates of treatment escalation and diagnostic testing compared with matched controls, although the magnitude of association varied by exposure group. This is represented graphically in Figure 2 below.

Patients with documented cannabis use were more likely to be admitted to the hospital (RR, 2.10; 95% CI, 1.72-2.56) and receive critical care services (RR, 1.71; 95% CI, 1.28-2.27). They also had higher rates of acute respiratory failure (RR, 2.68; 95% CI, 2.01-3.57) and were more frequently treated with systemic corticosteroids (RR, 1.22; 95% CI, 1.16-1.28), inhaled beta-agonists (RR, 1.30; 95% CI, 1.25-1.35), magnesium sulfate (RR, 2.15; 95% CI, 1.82-2.54), and CPAP (RR, 2.22; 95% CI, 1.42-3.49). Diagnostic testing was more common, including chest radiography (RR, 1.39; 95% CI, 1.28-1.49) and blood gas analysis (RR, 2.10; 95% CI, 1.65-2.67), and sedative administration occurred more frequently (RR, 1.59; 95% CI, 1.29-1.97).

Among patients with documented nicotine use, higher rates of hospital admission (RR, 1.55; 95% CI, 1.31-1.84) and acute respiratory failure (RR, 1.95; 95% CI, 1.54-2.47) were observed. These patients were more likely to receive systemic corticosteroids (RR, 1.23; 95% CI, 1.19-1.27), inhaled beta-agonists (RR, 1.27; 95% CI, 1.24-1.31), magnesium sulfate (RR, 1.48; 95% CI, 1.30-1.70), CPAP (RR, 1.57; 95% CI, 1.04-2.36), chest radiography (RR, 1.43, 95% CI, 1.36-1.51), blood gas testing (RR, 1.63; 95% CI, 1.34-1.98), and sedatives (RR, 1.28; 95% CI, 1.09-1.52). Rates of critical care services were not significantly different between groups (RR, 1.17; 95% CI, 0.89-1.53).

Among patients with documented co-use, associations were generally strongest, with higher risks of hospital admission (RR, 2.47; 95% CI, 1.80-3.38), critical care services (RR, 1.92; 95% CI, 1.19-3.09), and acute respiratory failure (RR, 4.63; 95% CI, 2.71-7.89). Co-use patients were also more likely to receive systemic corticosteroids (RR, 1.23; 95% CI, 1.14-1.33), inhaled beta-agonists (RR, 1.30; 95% CI, 1.22-1.38), magnesium sulfate (RR, 1.92; 95% CI, 1.49-2.47), chest radiography (RR, 1.44; 95% CI, 1.28-1.61), blood gas testing (RR, 2.34; 95% CI, 1.62-3.39), and sedatives (RR, 1.98; 95% CI, 1.41-2.78), while CPAP use was numerically higher but did not reach statistical significance (RR, 1.67; 95% CI, 0.88-3.14).

### 3.3 Outcomes From 1 day to 1 Month Postindex Visit

**Figure 3:**
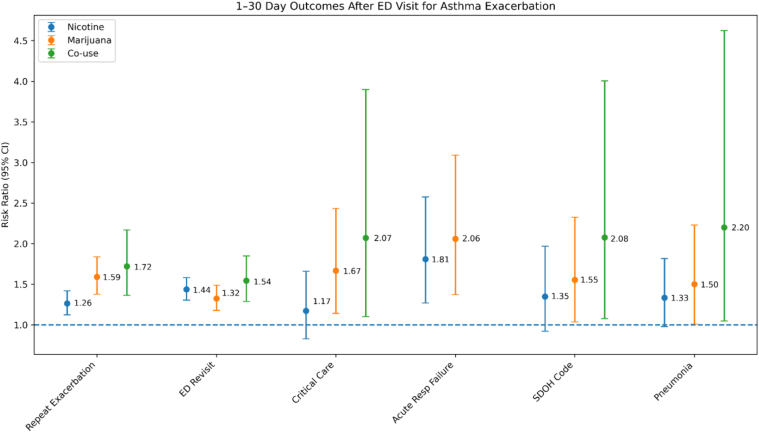
The 1- to 30-day outcomes after emergency department (ED) presentation for asthma exacerbation. Forest plot of risk ratios (RRs) with 95% CIs. Numeric RRs are displayed beside their respective datapoint; the dashed line indicates RR = 1.0.

Between 1 and 30 days following the index ED encounter, substance-specific cohorts continued to demonstrate higher rates of recurrent respiratory events and healthcare utilization compared with matched controls, with the strongest associations observed among patients with concurrent nicotine and cannabis use. This is represented graphically in Figure 3 below.

Among patients with documented cannabis use, there were higher rates of repeat asthma exacerbations (RR, 1.59; 95% CI, 1.38-1.84) and ED revisits (RR, 1.32; 95% CI, 1.18-1.49). They were also more likely to require critical care services (RR, 1.67; 95% CI, 1.14-2.43) and develop acute respiratory failure (RR, 2.06; 95% CI, 1.37-3.09). Additional associations included higher rates of pneumonia (RR, 1.50; 95% CI, 1.01-2.23) and assignment of social determinants of health-related diagnosis codes (RR, 1.55; 95% CI, 1.04-2.33).

With patients who had documented nicotine use, there was an increased risk of repeat asthma exacerbation (RR, 1.26; 95% CI, 1.12-1.42), ED revisits (RR, 1.44; 95% CI, 1.31-1.58), and acute respiratory failure (RR, 1.81; 95% CI, 1.27-2.58). Differences in critical care utilization, pneumonia, and social determinant-related diagnosis codes were not statistically significant.

Among patients with documented co-use, there were higher rates of repeat asthma exacerbation (RR, 1.72; 95% CI, 1.36-2.17) and ED revisits (RR, 1.54; 95% CI, 1.29-1.85), along with increased utilization of critical care services (RR, 2.07; 95% CI, 1.10-3.90). Co-use patients were also more likely to receive social determinant-related diagnosis codes (RR, 2.08; 95% CI, 1.08-4.00) and develop pneumonia (RR, 2.20; 95% CI, 1.05-4.62). Event counts for acute respiratory failure were suppressed because of small cell sizes.

## 4 Limitations

TriNetX aggregates deidentified EHR data from multiple health care organizations, and variability in documentation practices, coding behaviors, and institutional data transformation processes may introduce heterogeneity across sites. Exposure classification represents an additional limitation. Nicotine and cannabis use were identified using administrative diagnostic codes, which have demonstrated variable sensitivity and specificity across institutions, generally with high specificity but limited sensitivity. As a result, substance use may be underascertained, and identified patients may represent those with more clinically recognized or severe use. Additionally, important clinical variables including asthma severity, smoking or vaping patterns, secondhand smoke exposure, and outpatient medication adherence are not consistently captured in structured data, raising the potential for residual confounding. Our matching process was additionally unable to fully match for patient age, and although the degree of difference is unlikely to be clinically significant, this variable remains an important residual confounder. Outcome ascertainment was limited to coded encounters and procedures, and medication exposure was inferred from prescription or order records rather than confirmed administration, introducing potential measurement error. As with all retrospective EHR-based studies, these factors limit causal inference and may contribute to selection bias. In addition, the study assessed multiple outcomes across 3 exposure cohorts and 2 time windows without adjustment for multiple comparisons. As a result, some statistically significant findings, particularly those with CIs close to unity, may represent chance associations. Accordingly, these results should be interpreted as hypothesis-generating, and future studies are needed to confirm these findings in independent data sets.

We were unable to stratify analyses by ED type (eg, academic vs community) due to limitations in the granularity of site-level data available within the TriNetX platform. Variation in institutional practices may therefore influence the consistency of recorded variables, including measures of treatment intensity and diagnostic utilization. However, the large, multi-institutional nature of the data set and the use of PSM may help mitigate, though not eliminate, the impact of site-level variability. Finally, the extended study period (2015-2025) spans substantial changes in adolescent substance use patterns, including increasing cannabis legalization and the rapid rise of e-cigarette use. Vaping represents a distinct form of nicotine exposure compared with combustible tobacco, with differences in aerosol composition and particulate exposure that may differentially affect airway biology. However, ICD-10 coding within the TriNetX platform does not allow reliable differentiation between vaping and traditional combustible nicotine use. As a result, the nicotine exposure variable reflects heterogeneous modalities of use, which may have differing effects on asthma pathophysiology and exacerbation severity. Temporal changes in both substance use patterns and clinical documentation practices may further introduce heterogeneity and exposure misclassification, particularly in earlier years when vaping-related diagnoses were less consistently recorded. These factors should be considered when interpreting the observed associations.

## 5 Discussion

### 5.1 Key Results

This retrospective cohort study found that adolescents were more likely to have higher rates of treatment escalation and diagnostic testing while presenting to the ED if they had a documented nicotine and/or cannabis use disorder when compared to matched controls. Across all 3 groups, patients more frequently received inhaled beta-agonists, systemic corticosteroids, and magnesium sulfate, underwent chest radiography and blood gas analysis, and were more likely to be admitted to the hospital. Associations were generally strongest among patients with concurrent documented nicotine and cannabis use.

During the subsequent 1 to 30 days, all exposure cohorts experienced higher rates of repeated asthma exacerbations and ED revisits. Patients with documented cannabis use and co-use additionally demonstrated increased risks of critical care utilization, pneumonia, and assignment of social determinant-related diagnosis codes, whereas patients with only documented nicotine use showed elevated risks of recurrent exacerbation and acute respiratory failure but not of later critical care use. Overall, these findings identify adolescents with documented nicotine and/or cannabis use disorder diagnoses, particularly those with co-use, as a subgroup with more intensive acute management and higher short-term utilization following asthma exacerbations.

### 5.2 Analysis

Our key findings largely agree and supplement existing literature on the influence of concurrent substance use disorder in adolescents with asthma exacerbations. The graded pattern observed across exposure groups, with the strongest associations among patients with co-use, is consistent with emerging evidence that cannabis use is associated with increased asthma prevalence in a dose-dependent manner and that combined tobacco and cannabis exposure may confer additive respiratory risk.^10^^,^^21^ Our study has specifically shown that this additive respiratory risk may manifest as greater acute severity at ED presentation compared to their non-comorbid peers. This scenario is identified partly through our finding that co-use is associated with a relative risk of 4.63 (95% CI, 2.71-7.89) for acute respiratory failure, compared with tobacco alone (RR, 1.95, 95% CI, 1.54-2.47) or cannabis alone (RR, 2.68, 95% CI, 2.01-3.57).

Existing studies linking substance use to asthma morbidity have often focused on downstream outcomes such as intubation, intensive care unit (ICU) admission, or length of stay.^22^, ^23^, ^24^ Looking acutely at how these patients are presenting to the ED, we are able to see that they require more immediate pharmacologic therapy, noninvasive ventilation, radiography, and diagnostic testing when compared to their peers at the point of acute care. These findings suggest that identification of substance use disorder may help risk-stratify adolescents presenting with asthma exacerbation. This is supported by existing literature showing that cannabis and tobacco smoking are each associated with worse respiratory symptoms such as cough, sputum production, wheezing, and dyspnea.^11^^,^^24^^,^^25^

GINA guidelines emphasize that active smoking is associated with increased risk of poor asthma control, hospital admission, and reduced responsiveness to corticosteroid therapy, while growing evidence links vaping to increased risk of exacerbations.^15^ Our finding that adolescents with nicotine and/or cannabis use were more likely to re-present to the ED within 30 days suggests persistent disease instability beyond the index exacerbation. Several mechanisms may explain this association. Ongoing exposure to inhaled irritants can perpetuate airway inflammation and bronchial hyperresponsiveness, while tobacco smoke has been associated with impaired corticosteroid responsiveness, potentially limiting the effectiveness of standard controller and rescue therapies.^26^ Cannabis and nicotine use may also contribute to mucus hypersecretion and airway inflammation, while inhalational exposures associated with smoking or vaping (including particulate matter and aerosols) can promote epithelial injury, further exacerbating airflow limitation.^26^ In addition, behavioral factors such as medication nonadherence, reduced engagement with outpatient follow-up, and coexisting psychosocial stressors may impair post-discharge recovery.^12^ Together, these biological and behavioral pathways may predispose this population to recurrent exacerbations and short-term health care reutilization.

Psychiatric comorbidity is a well-established contributor to asthma morbidity in pediatric populations. Anxiety and depressive disorders have consistently been associated with increased asthma-related ED utilization, longer hospitalizations, higher health care costs, and poorer symptom control.^27^, ^28^, ^29^ Anxiety, in particular, has been linked to worse asthma-related quality of life and distorted perception of symptom severity.^30^ In our study, prior to PSM, adolescents with nicotine and/or cannabis-related diagnoses had substantially higher rates of comorbid mental health conditions. This imbalance suggests that psychiatric comorbidity may represent an important confounder in the relationship between substance use and asthma severity. Because our matching process equalized rates of mental health diagnoses across cohorts, the independent effect of psychiatric comorbidity on outcomes was not evaluated. It is plausible that psychiatric disorders and substance use disorder exert synergistic effects on asthma morbidity; therefore, our findings may underestimate the overall burden of disease experienced by adolescents in whom these conditions coexist.

From a clinical standpoint, these findings reinforce existing recommendations to integrate substance-use screening and behavioral health assessment into routine adolescent care. The American Academy of Pediatrics endorses universal screening using validated tools such as Car, Relax, Alone, Forget, Friends, Trouble (CRAFFT) or Screening to Brief Intervention (S2BI) screening tool, followed by brief intervention and referral to treatment (SBIRT).^31^^,^^32^ Growing evidence suggests that brief interventions delivered in clinical settings, including EDs, can reduce substance use and improve linkage to treatment.^33^ Whether such strategies can modify acute management intensity or short-term outcomes among adolescents with asthma presenting to the ED warrants further prospective investigation.

Adolescents with documented use of nicotine, cannabis, and concurrent nicotine-cannabis substances experienced greater health care utilization and treatment intensity when presenting with asthma exacerbations, both during the index ED encounter and in the subsequent month. These findings suggest an opportunity to develop targeted ED care pathways for adolescents with asthma and coexisting substance use. Potential components of such pathways include standardized screening for nicotine and cannabis use at triage, incorporation of substance use status into risk stratification for exacerbation severity, earlier escalation of therapy or observation for higher-risk patients, and integration of brief intervention strategies (eg, counseling or referral to cessation resources) prior to discharge. Structured discharge planning with close outpatient follow-up may further help reduce recurrent exacerbations and repeat ED utilization in this population. Future studies should examine long-term respiratory and psychosocial outcomes and evaluate whether ED-based interventions can mitigate recurrent exacerbations and health care utilization among adolescents with asthma and substance-related diagnoses.

## Author Contribution

HS: Writing - review & editing, Writing - original draft, Methodology, Investigation, Data curation, Conceptualization.

AM: Writing - review & editing, Writing - original draft, Methodology, Investigation, Data curation, Conceptualization.

SR: Writing - review & editing, Writing - original draft.

SS: Writing - review& editing, Supervision, Methodology, Investigation, Conceptualization.

## Conflict of Interest

The authors declare that they have no known competing financial interests or personal relationships that could have appeared to influence the work reported in this paper.
