# Supplementary material for: Documented Nicotine, Cannabis, and Co-Use Are Associated With More Intensive Emergency Department Care for Adolescent Asthma Exacerbations
Source: J Am Coll Emerg Physicians Open. 2026 Jun 29;7(4):100453. doi: 10.1016/j.acepjo.2026.100453 (PMC13331984; doi:10.1016/j.acepjo.2026.100453)
Supplement: Supplementary Tables 1-6 [file mmc1.docx]

| Short-Term Outcomes of Interest | Coding Systems and Codes Used |
| --- | --- |
| Hospital admission from ED | CPT: 1013661, 1013675 |
| Critical care services | CPT: 99291, 99292 |
| Acute respiratory failure | ICD-10: J96.0 |
| Administration of systemic corticosteroids | RxNorm: 8640 (prednisone), 3264 (dexamethasone), 8638 (prednisolone) |
| Administration of inhaled beta agonists | RxNorm: 435 (albuterol), 237159 (levalbuterol) |
| Magnesium sulfate administration | RxNorm: 6585 (Magnesium sulfate) |
| CPAP Use | CPT: 94660  ICD-10: 5A09357, 5A09458, 5A09557 |
| Chest X-Ray | CPT 1014245, 1014248, 1031050 |
| Blood gas analysis | CPT 1014265 |
| Sedative use | RxNorm: 6960 (midazolam), 6130 (ketamine), 6470 (lorazepam), 5093 (haloperidol), 3322 (diazepam), 8745 (promethazine), 115698 (ziprasidone), 3648 (droperidol), 61382 (olanzapine) |
| Social determinant of health | ICD-10: Z55 (education and literacy), Z56 (employment and unemployment), Z57 (occupational exposure), Z59 (housing and economic circumstances), Z60 (social environment), Z62 (upbringing), Z63 (primary support group, including family circumstances), Z64 (psychosocial circumstances), and Z65 (social exclusion and rejection) |

**Supplemental Table S1**. Short-term outcomes of interest with their associated coding definition.

| **Nicotine vs non-SUD cohort** | | **Pre-Propensity Score Matching** | | | | **Post-Propensity Score Matching** | | | |
| --- | --- | --- | --- | --- | --- | --- | --- | --- | --- |
| **Demographics** | | **Nicotine cohort n (%)** | **Non-SUD cohort n (%)** | **P value** | **Std. Diff** | **Nicotine cohort n (%)** | **Non-SUD cohort n (%)** | **P value** | **Std. Diff** |
| **Age at index, mean ± SD (yrs)** | | 19.3 ± 2.21 | 15.7 ± 3.61 | <0.001 | 1.2330 | 19.3 ± 2.21 | 18.1 ± 2.55 | <0.001 | 0.5061 |
| **Age categories** | **Age 5-10 yrs** | 0 (0%) | 0 (0%) | - | - | 0 (0%) | 0 (0%) | - | - |
|  | **Age 10-15 yrs** | 143 (3.10%) | 30,074 (43.51%) | <0.001 | 1.0879 | 143 (3.11%) | 143 (3.11%) | 1.00 | <0.001 |
|  | **Age 15-21 yrs** | 4,207 (91.32%) | 36,335 (52.56%) | <0.001 | 0.9560 | 4,204 (91.31%) | 4,218 (91.62%) | 0.602 | 0.0109 |
| **Gender** | **Female** | 2,549 (55.33%) | 35,415 (51.23%) | <0.001 | 0.0822 | 2,549 (55.37%) | 2,574 (55.91%) | 0.600 | 0.0109 |
|  | **Male** | 2,058 (44.67%) | 33,711 (48.77%) | <0.001 | 0.0822 | 2,055 (44.64%) | 2,030 (44.09%) | 0.600 | 0.0109 |
| **Race** | **White** | 2,345 (50.90%) | 28,322 (40.97%) | <0.001 | 0.2002 | 2,343 (50.89%) | 2,354 (51.13%) | 0.819 | 0.0048 |
|  | **Black/African American** | 1,682 (36.51%) | 27,864 (40.31%) | <0.001 | 0.0782 | 1,681 (36.51%) | 1,695 (36.82%) | 0.762 | 0.0063 |
|  | **Asian** | 77 (1.67%) | 1,757 (2.54%) | 0.0002 | 0.0606 | 77 (1.67%) | 81 (1.76%) | 0.748 | 0.0067 |
| **Ethnicity** | **Hispanic/Latino** | 485 (10.53%) | 12,149 (17.58%) | <0.001 | 0.2039 | 485 (10.53%) | 480 (10.43%) | 0.865 | 0.0035 |
|  | **Not Hispanic/Latino** | 3,167 (68.74%) | 44,910 (64.97%) | <0.001 | 0.0803 | 3,164 (68.72%) | 3,166 (68.77%) | 0.964 | 0.0009 |
|  | **Unknown Ethnicity** | 955 (20.73%) | 12,067 (17.46%) | <0.001 | 0.0833 | 955 (20.74%) | 958 (20.81%) | 0.939 | 0.0016 |
| **Diagnosis (ICD-10 category)** | | **Nicotine cohort n (%)** | **Non-SUD cohort n (%)** | **P value** | **Std. Diff** | **Nicotine cohort n (%)** | **Non-SUD cohort n (%)** | **P value** | **Std. Diff** |
| **Chronic lower respiratory disease (J40-J4A)** | | 3,383 (73.43%) | 50,331 (72.81%) | 0.359 | 0.0140 | 3,380 (73.41%) | 3,366 (73.11%) | 0.742 | 0.0069 |
| **Genitourinary disease (N00-N99)** | | 1,895 (41.13%) | 17,928 (25.94%) | <0.001 | 0.3262 | 1,892 (41.10%) | 1,884 (40.92%) | 0.865 | 0.0035 |
| **Mood disorders (F30-F39)** | | 1,725 (37.44%) | 8,385 (12.13%) | <0.001 | 0.6132 | 1,722 (37.40%) | 1,706 (37.06%) | 0.730 | 0.0072 |
| **Anxiety disorders (F40-F48)** | | 1,712 (37.16%) | 12,296 (17.79%) | <0.001 | 0.4446 | 1,709 (37.12%) | 1,713 (37.21%) | 0.931 | 0.0018 |
| **Behavioral childhood disorders (F90-F98)** | | 1,041 (22.60%) | 10,301 (14.90%) | <0.001 | 0.1981 | 1,038 (22.55%) | 1,003 (21.79%) | 0.380 | 0.0183 |
| **Overweight/obesity (E65-E68)** | | 935 (20.30%) | 11,182 (16.18%) | <0.001 | 0.1068 | 934 (20.29%) | 925 (20.09%) | 0.815 | 0.0049 |
| **Other intestinal disease (K55-K64)** | | 840 (18.23%) | 12,176 (17.61%) | 0.286 | 0.0161 | 839 (18.22%) | 816 (17.72%) | 0.533 | 0.0130 |
| **Metabolic disorders (E70-E88)** | | 805 (17.47%) | 9,725 (14.07%) | <0.001 | 0.0935 | 803 (17.44%) | 759 (16.49%) | 0.222 | 0.0255 |
| **Neoplasms (C00-D49)** | | 230 (4.99%) | 3,013 (4.36%) | 0.042 | 0.0300 | 229 (4.97%) | 210 (4.56%) | 0.353 | 0.0194 |
| **Developmental disorders (F80-F89)** | | 207 (4.49%) | 5,377 (7.78%) | <0.001 | 0.1372 | 207 (4.50%) | 185 (4.02%) | 0.256 | 0.0237 |
| **MSK malformations (Q65-Q79)** | | 117 (2.54%) | 2,531 (3.66%) | <0.001 | 0.0648 | 117 (2.54%) | 94 (2.04%) | 0.109 | 0.0334 |
| **Coagulation defects (D65-D69)** | | 82 (1.78%) | 937 (1.36%) | 0.017 | 0.0342 | 82 (1.78%) | 65 (1.41%) | 0.158 | 0.0295 |
| **Congenital heart disease (Q20-Q28)** | | 55 (1.19%) | 1,447 (2.09%) | <0.001 | 0.0708 | 55 (1.20%) | 37 (0.80%) | 0.059 | 0.0393 |
| **Other conduction disorders (I45)** | | 52 (1.13%) | 426 (0.62%) | <0.001 | 0.0551 | 51 (1.11%) | 26 (0.57%) | 0.004 | 0.0597 |
| **Sickle cell disease (D57)** | | 49 (1.06%) | 723 (1.05%) | 0.909 | 0.0017 | 49 (1.06%) | 48 (1.04%) | 0.919 | 0.0021 |
| **Cardiomyopathy (I42)** | | 18 (0.39%) | 184 (0.27%) | 0.117 | 0.0218 | 16 (0.35%) | 10 (0.22%) | 0.239 | 0.0246 |
| **Chromosomal abnormalities (Q90-Q99)** | | 10 (0.22%) | 530 (0.77%) | <0.001 | 0.0786 | 10 (0.22%) | 10 (0.22%) | 1.00 | <0.001 |

**Supplemental Table S2**. Baseline demographics between Nicotine-only group and non-substance use disorder group.

| **Cannabis vs non-SUD cohort** | | | **Pre-Propensity Score Matching** | | | | **Post-Propensity Score Matching** | | |
| --- | --- | --- | --- | --- | --- | --- | --- | --- | --- |
| **Demographics**  **Pre-Match cannabis n (%)** | | | **Cannabis cohort n (%)** | **Non-SUD cohort n (%)** | **P value** | **Std. Diff** | **Cannabis cohort n (%)** | **Non-SUD cohort n (%)** | **P value** |
| **Age at index, mean ± SD (yrs)** | | 18.9 ± 2.2 | 15.8 ± 3.64 | <0.001 | 1.0438 | 18.9 ± 2.2 | 18.2 ± 2.48 | <0.001 | 0.3219 |
| **Age** | **Age 5-10 yrs** | 0 (0%) | 0 (0%) | - | - | 0 (0%) | 0 (0%) | - | - |
|  | **Age 10-15 yrs** | 67 (2.50%) | 30,118 (42.39%) | <0.001 | 1.0885 | 67 (2.50%) | 68 (2.54%) | 0.931 | 0.0024 |
|  | **Age 15-21 yrs** | 2,486 (92.73%) | 38,102 (53.63%) | <0.001 | 0.9835 | 2,484 (92.72%) | 2,487 (92.83%) | 0.874 | 0.0043 |
| **Gender** | **Female** | 1,471 (54.87%) | 36,493 (51.36%) | 0.0004 | 0.0703 | 1,471 (54.91%) | 1,474 (55.02%) | 0.934 | 0.0023 |
|  | **Male** | 1,210 (45.13%) | 34,559 (48.64%) | 0.0004 | 0.0703 | 1,208 (45.09%) | 1,205 (44.98%) | 0.934 | 0.0023 |
| **Race** | **White** | 1,159 (43.23%) | 29,508 (41.53%) | 0.080 | 0.0344 | 1,159 (43.26%) | 1,173 (43.79%) | 0.700 | 0.0105 |
|  | **Black/African American** | 1,112 (41.48%) | 28,434 (40.02%) | 0.130 | 0.0297 | 1,110 (41.43%) | 1,121 (41.84%) | 0.761 | 0.0083 |
|  | **Asian** | 34 (1.27%) | 1,800 (2.53%) | <0.001 | 0.0928 | 34 (1.27%) | 34 (1.27%) | 1.00 | <0.001 |
| **Ethnicity** | **Hispanic/Latino** | 446 (16.64%) | 12,188 (17.15%) | 0.485 | 0.0138 | 445 (16.61%) | 425 (15.86%) | 0.459 | 0.0202 |
|  | **Not Hispanic/Latino** | 1,803 (67.25%) | 46,274 (65.13%) | 0.023 | 0.0449 | 1,802 (67.26%) | 1,830 (68.31%) | 0.413 | 0.0224 |
|  | **Unknown Ethnicity** | 432 (16.11%) | 12,590 (17.72%) | 0.032 | 0.0428 | 432 (16.13%) | 424 (15.83%) | 0.766 | 0.0082 |
| **Diagnosis (ICD-10 category)** | | **Cannabis cohort n (%)** | **Non-SUD cohort n (%)** | **P value** | **Std. Diff** | **Cannabis cohort n (%)** | **Non-SUD cohort n (%)** | **P value** | **Std. Diff** |
| **Chronic lower respiratory disease (J40-J4A)** | | 2,225 (82.99%) | 51,443 (72.40%) | <0.001 | 0.2565 | 2,223 (82.98%) | 2,232 (83.32%) | 0.743 | 0.0090 |
| **Anxiety disorders (F40-F48)** | | 1,297 (48.38%) | 12,722 (17.91%) | <0.001 | 0.6842 | 1,295 (48.34%) | 1,295 (48.34%) | 1.00 | <0.001 |
| **Mood disorders (F30-F39)** | | 1,287 (48.00%) | 8,820 (12.41%) | <0.001 | 0.8408 | 1,285 (47.97%) | 1,274 (47.56%) | 0.764 | 0.0082 |
| **Genitourinary disease (N00-N99)** | | 1,183 (44.13%) | 18,613 (26.20%) | <0.001 | 0.3823 | 1,183 (44.16%) | 1,202 (44.87%) | 0.602 | 0.0143 |
| **Behavioral childhood disorders (F90-F98)** | | 775 (28.91%) | 10,567 (14.87%) | <0.001 | 0.3444 | 773 (28.85%) | 758 (28.29%) | 0.650 | 0.0124 |
| **Metabolic disorders (E70-E88)** | | 651 (24.28%) | 9,889 (13.92%) | <0.001 | 0.2660 | 650 (24.26%) | 655 (24.45%) | 0.874 | 0.0043 |
| **Overweight/obesity (E65-E68)** | | 605 (22.57%) | 11,503 (16.19%) | <0.001 | 0.1619 | 604 (22.55%) | 600 (22.40%) | 0.896 | 0.0036 |
| **Other intestinal disease (K55-K64)** | | 586 (21.86%) | 12,426 (17.49%) | <0.001 | 0.1101 | 586 (21.87%) | 570 (21.28%) | 0.595 | 0.0145 |
| **Neoplasms (C00-D49)** | | 167 (6.23%) | 3,075 (4.33%) | <0.001 | 0.0851 | 167 (6.23%) | 149 (5.56%) | 0.297 | 0.0285 |
| **Developmental disorders (F80-F89)** | | 143 (5.33%) | 5,445 (7.66%) | <0.001 | 0.0946 | 143 (5.34%) | 132 (4.93%) | 0.496 | 0.0186 |
| **MSK malformations (Q65-Q79)** | | 79 (2.95%) | 2,568 (3.61%) | 0.068 | 0.0375 | 79 (2.95%) | 75 (2.80%) | 0.744 | 0.0089 |
| **Coagulation defects (D65-D69)** | | 55 (2.05%) | 958 (1.35%) | 0.002 | 0.0544 | 55 (2.05%) | 60 (2.24%) | 0.637 | 0.0129 |
| **Sickle-cell disease (D57)** | | 55 (2.05%) | 722 (1.02%) | <0.001 | 0.0843 | 53 (1.98%) | 39 (1.46%) | 0.141 | 0.0402 |
| **Other conduction disorders (I45)** | | 41 (1.53%) | 442 (0.62%) | <0.001 | 0.0880 | 41 (1.53%) | 34 (1.27%) | 0.416 | 0.0222 |
| **Congenital heart disease (Q20-Q28)** | | 32 (1.19%) | 1,469 (2.07%) | 0.002 | 0.0690 | 32 (1.19%) | 24 (0.90%) | 0.283 | 0.0294 |
| **Cardiomyopathy (I42)** | | 11 (0.41%) | 190 (0.27%) | 0.164 | 0.0246 | 11 (0.41%) | 10 (0.37%) | 0.827 | 0.0060 |
| **Chromosomal abnormalities (Q90-Q99)** | | 10 (0.37%) | 531 (0.75%) | 0.026 | 0.0502 | 10 (0.37%) | 10 (0.37%) | 1.00 | <0.001 |

**Supplemental Table S3**. Baseline demographics between cannabis-only group and non-substance use disorder group.

| **CoUse vs non-SUD cohort** | | **Pre-Propensity Score Matching** | | | | **Post-Propensity Score Matching** | | | |
| --- | --- | --- | --- | --- | --- | --- | --- | --- | --- |
| **Demographics** | | **SUD cohort n (%)** | **Non-SUD cohort n (%)** | **P value** | **Std. Diff** | **SUD cohort n (%)** | **Non-SUD cohort n (%)** | **P value** | **Std. Diff** |
|  | Age at index, mean ± SD (years) | 19.3 ± 1.98 | 15.6 ± 3.6 | <0.001 | 1.27 | 19.3 ± 1.99 | 18.1 ± 2.3 | <0.001 | 0.56 |
| Age Categories | 5-10 years | 0 |  |  |  |  |  |  |  |
|  | 10-15 years | 10 (0.954%) | 29,512 (44.489%) | <0.001 | 1.22 | 10 (0.957%) | 10 (0.957%) | 1 | <0.01 |
|  | 15-21 years | 993 (94.752%) | 34,206 (51.565%) | <0.001 | 1.12 | 990 (94.737%) | 998 (95.502%) | 0.4167 | 0.0355 |
| Gender | Female | 557 (53.149%) | 33,847 (51.024%) | 0.1721 | 0.0436 | 557 (53.301%) | 562 (53.78%) | 0.8264 | 0.0096 |
|  | Male | 491 (46.851%) | 32,489 (48.976%) | 0.1721 | 0.0426 | 488 (46.699%) | 482 (46.22%) | 0.8264 | 0.0096 |
| Race | White | 531 (50.668%) | 27,020 (40.732%) | <0.001 | 0.2005 | 530 (50.718%) | 527 (50.431%) | 0.8956 | 0.0057 |
|  | Black or African American | 392 (37.405%) | 26,733 (40.299%) | 0.0580 | 0.0594 | 391 (37.416%) | 410 (39.234%) | 0.3926 | 0.0374 |
|  | Asian | 13 (1.24%) | 1,731 (2.609%) | 0.0056 | 0.0998 | 13 (1.244%) | 13 (1.244%) | 1.0 | <0.001 |
| Ethnicity | Hispanic or Latino | 133 (12.691%) | 11,776 (17.752%) | <0.0001 | 0.1412 | 133 (12.727%) | 119 (11.388%) | 0.3470 | 0.0412 |
|  | Not Hispanic or Latino | 728 (69.466%) | 42,760 (64.46%) | 0.0008 | 0.1066 | 725 (69.378%) | 735 (70.335%) | 0.6336 | 0.0209 |
|  | Unknown ethnicity | 187 (17.844%) | 11,800 (17.788%) | 0.9630 | 0.0014 | 187 (17.895%) | 191 (18.278%) | 0.8202 | 0.0099 |
| **Diagnosis (ICD-10 category)** | | **SUD cohort n (%)** | **Non-SUD cohort n (%)** | **P value** | **Std. Diff** | **SUD cohort n (%)** | **Non-SUD cohort n (%)** | **P value** | **Std. Diff** |
| Mood disorders (F30-F39) | | 606 (57.824%) | 7,689 (11.591%) | <0.0001 | 1.1110 | 603 (57.703%) | 595 (56.938%) | 0.7235 | 0.0155 |
| Anxiety & stress-related disorders (F40-F48) | | 597 (56.966%) | 11,537 (17.392%) | <0.0001 | 0.8975 | 594 (56.842%) | 595 (56.938%) | 0.9648 | 0.0019 |
| Behavioral disorders of childhood (F90-F98) | | 359 (34.256%) | 9,839 (14.832%) | <0.0001 | 0.4633) | 356 (34.067%) | 359 (34.354%) | 0.8900 | 0.0061 |
| Developmental disorders (F80-F89) | | 62 (5.916%) | 5,279 (7.958%) | 0.0152 | 0.0804 | 62 (5.933%) | 66 (6.316%) | 0.7152 | 0.0160 |
| Overweight/obesity (E65-E68) | | 261 (24.905%) | 10,822 (16.314%) | <0.0001 | 0.2136 | 259 (24.785%) | 258 (24.689%) | 0.9596 | 0.0022 |
| Metabolic disorders (E70-E88) | | 286 (27.29%) | 9,385 (14.148%) | <0.0001 | 0.3286 | 283 (27.081%) | 273 (26.124%) | 0.6206 | 0.0217 |
| Genitourinary disease (N00-N99) | | 508 (48.473%) | 17,145 (25.846%) | <0.0001 | 0.4816 | 505 (48.325%) | 514 (49.187%) | 0.6937 | 0.0172 |
| Chronic lower respiratory disease (J40-J4A) | | 896 (85.496%) | 48,590 (73.248%) | <0.0001 | 0.3062 | 893 (85.455%) | 901 (86.22%) | 0.6157 | 0.0220 |
| Congenital heart disease (Q20-Q28) | | 18 (1.718%) | 1,426 (2.15%) | 0.3378 | 0.0314 | 18 (1.722%) | 11 (1.053%) | 0.1905 | 0.0573 |
| Sickle-cell disease (D57) | | 25 (2.385%) | 692 (1.043%) | <0.0001 | 0.1035 | 24 (2.297%) | 21 (2.01%) | 0.6512 | 0.0198 |
| Other diseases of the intestines (K55-K64 | | 263 (25.095%) | 11,808 (17.8%) | <0.0001 | 0.1784 | 261 (24.976%) | 255 (24.402%) | 0.7608 | 0.0133 |
| Neoplasms (C00-D49) | | 80 (7.634%) | 2,912 (4.39%) | <0.0001 | 0.1368 | 78 (7.464%) | 59 (5.646%) | 0.0931 | 0.0735 |
| Congenital malformations of the musculoskeletal system (D65-D79) | | 39 (3.721%) | 2,478 (3.736%) | 0.9809 | 0.0007 | 38 (3.636%) | 30 (2.871%) | 0.3240 | 0.0432 |
| Coagulation defects, purpura, and other hematologic conditions (D65-D69) | | 26 (2.481%) | 900 (1.357%) | 0.0019 | 0.0820 | 25 (2.392%) | 14 (1.34%) | 0.0754 | 0.0778 |
| Other conduction disorders (I45) | | 21 (2.004%) | 408 (0.615%) | <0.0001 | 0.1224 | 21 (2.01%) | 14 (1.34%) | 0.2328 | 0.0522 |
| Chromosomal abnormalities, not otherwise specified (Q90-Q99) | | 10 (0.954%) | 526 (0.793%) | 0.5598 | 0.0173 | 10 (0.957%) | 10 (0.957%) | 1.00 | <0.0001 |
| Cardiomyopathy (I42) | | 10 (0.954%) | 183 (0.276%) | <0.0001 | 0.0868 | 10 (0.957%) | 10 (0.957%) | 1.00 | <0.0001 |

**Supplemental Table S4**. Baseline demographics between cannabis and Nicotine group and non-substance use disorder group.

| **0 Day to 1 Day Analysis** | **Asthma + Nicotine** | **Asthma - Nicotine** | **Chi-Square p value** | **Risk ratio (95% CI)** |
| --- | --- | --- | --- | --- |
| Hospital admission from ED | 326 (7.081%) | 210 (4.561%) | <0.0001 | 1.552 (1.312, 1.837) |
| Critical Care Services | 112 (2.433%) | 96 (2.085%) | 0.2618 | 1.167 (0.891, 1.528) |
| Acute respiratory failure | 195 (4.235%) | 100 (2.172%) | <0.0001 | 1.95 (1.538, 2.473) |
| Administration of systemic corticosteroids | 2,941 (63.879%) | 2,392 (51.955%) | <0.0001 | 1.23 (1.187, 1.274) |
| Administration of inhaled beta-agonist | 2,605 (78.301%) | 2,839 (61.664%) | <0.0001 | 1.27 (1.236, 1.305) |
| Magnesium sulfate administration | 478 (10.382%) | 322 (6.994%) | <0.0001 | 1.484 (1.297, 1.699) |
| CPAP initiation | 58 (1.26%) | 37 (0.804%) | 0.0303 | 1.568 (1.04, 2.363) |
| Chest X-ray | 1,995 (43.332%) | 1,394 (30.278%) | <0.0001 | 1.431 (1.355, 1.512) |
| Blood gas analysis | 257 (5.582%) | 158 (3.432%) | <0.0001 | 1.627 (1.34, 1.975) |
| Sedative | 295 (6.407%) | 230 (4.996%) | 0.0035 | 1.283 (1.085, 1.516) |
| **0 Day to 1 Day Analysis** | **Asthma + cannabis** | **Asthma - cannabis** | **Chi-Square p value** | **Risk ratio (95% CI)** |
| Hospital admission from ED | 283 (10.6%) | 135 (5.039%) | <0.001 | 2.096 (1.72, 2.56) |
| Critical Care Services | 123 (4.59%) | 72 (2.69%) | 0.0002 | 1.708 (1.284, 2.274) |
| Acute respiratory failure | 166 (6.196%) | 62 (2.314%) | <0.001 | 2.677 (2.01, 3.567) |
| Administration of systemic corticosteroids | 1,644 (61.366%) | 1,347 (50.28%) | <0.0001 | 1.22 (1.163, 1.281) |
| Administration of inhaled beta-agonist | 2,068 (77.193%) | 1,589 (59.313%) | <0.0001 | 1.301 (1.254, 1.351) |
| Magnesium sulfate administration | 389 (14.52%) | 181 (6.756%) | <0.0001 | 2.149 (1.817, 2.542) |
| CPAP initiation | 60 (2.24%) | 27 (1.008%) | 0.0004 | 2.222 (1.415, 3.489) |
| Chest X-ray | 1,062 (39.642%) | 767 (28.63%) | <0.0001 | 1.385 (1.283, 1.494) |
| Blood gas analysis | 195 (7.279%) | 93 (3.471%) | <0.0001 | 2.097 (1.648, 2.669) |
| Sedative | 212 (7.931%0 | 133 (4.965%) | <0.0001 | 1.594 (1.292, 1.967) |
| **0 Day to 1 Day Analysis** | **Asthma + Co-use** | **Asthma - Co-use** | **Chi-Square p value** | **Risk ratio (95% CI)** |
| Hospital admission from ED | 126 (11.549%) | 51 (4.675%) | <0.0001 | 2.471 (1.804, 3.383) |
| Critical Care Services | 48 (4.4%) | 25 (2.291%) | 0.0062 | 1.92 (1.193, 3.091) |
| Acute respiratory failure | 74 (6.783%) | 16 (1.467%) | <0.0001 | 4.625 (2.712, 7.888) |
| Administration of systemic corticosteroids | 654 (59.945%) | 532 (48.763%) | <0.0001 | 1.229 (1.137, 1.329) |
| Administration of inhaled beta-agonist | 830 (76.077%) | 640 (58.662%) | <0.0001 | 1.297 (1.221, 1.377) |
| Magnesium sulfate administration | 157 (14.39%) | 82 (7.516%) | <0.0001 | 1.915 (1.486, 2.467) |
| CPAP initiation | 25 (2.291%) | 15 (1.375%) | 0.1105 | 1.667 (0.884, 3.144) |
| Chest X-ray | 472 (43.263%) | 329 (30.156%) | <0.0001 | 1.435 (1.281, 1.606) |
| Blood gas analysis | 89 (8.158%) | 38 (3.483%) | <0.0001 | 2.342 (1.617, 3.392) |
| Sedative | 93 (8.524%) | 47 (4.308%) | <0.0001 | 1.979 (1.408, 2.782) |

**Supplemental Table S5**. 0-1 day analysis between Cases and Controls.

| **1 Day to 1 Month Analysis** | **Asthma + Nicotine** | **Asthma - Nicotine** | **Chi-Square p value** | **Risk ratio (95% CI)** |
| --- | --- | --- | --- | --- |
| Repeat asthma exacerbation | 562 (12.207%) | 445 (9.666%) | <0.0001 | 1.263 (1.123, 1.42) |
| Emergency department services | 855 (18.571%) | 595 (12.924%) | <0.0001 | 1.437 (1.305, 1.582) |
| Critical care services | 68 (1.477%) | 58 (1.26%) | 0.3697 | 1.172 (0.828, 1.66) |
| Acute respiratory failure | 85 (1.846%) | 47 (1.021%) | 0.0009 | 1.809 (1.269, 2.576) |
| Social determinant of health code assignment | 62 (1.347%) | 46 (0.999%) | 0.1215 | 1.348 (0.922, 1.969) |
| Pneumonia | 92 (1.998%) | 69 (1.499%) | 0.0674 | 1.333 (0.978, 1.817) |
| **1 Day to 1 Month Analysis** | **Asthma + cannabis** | **Asthma - cannabis** | **Chi-Square p value** | **Risk ratio (95% CI)** |
| Repeat asthma exacerbation | 420 (15.677%) | 264 (9.854%) | <0.0001 | 1.591 (1.377, 1.838) |
| Emergency department services | 529 (20.119%) | 407 (15.192%) | <0.0001 | 1.324 (1.178, 1.489) |
| Critical care services | 70 (2.613%) | 42 (1.568%) | 0.0075 | 1.667 (1.141, 2.434) |
| Acute respiratory failure | 70 (2.613%) | 34 (1.269%) | 0.0004 | 2.059 (1.372, 3.091) |
| Social determinant of health code assignment | 59 (2.202%) | 38 (1.418%) | 0.0314 | 1.553 (1.036, 2.326) |
| Pneumonia | 60 (2.24%) | 40 (1.492%) | 0.0435 | 1.5 (1.009, 2.23) |
| **1 Day to 1 Month Analysis** | **Asthma + Co-Use** | **Asthma-Co-use** | **Chi-Square p value** | **Risk ratio (95% CI)** |
| Repeat asthma exacerbation | 172 (15.765%) | 100 (9.166%) | <0.0001 | 1.72 (1.364, 2.169) |
| Emergency department services | 247 (22.64%) | 160 (14.665%) | <0.0001 | 1.544 (1.289, 1.849) |
| Critical care services | 29 (2.658%) | 14 (1.283%) | 0.0209 | 2.071 (1.101, 3.898) |
| Acute respiratory failure | <10 | <10 | NA | NA |
| Social determinant of health code assignment | 27 (2.475%) | 13 (1.192%) | 0.0255 | 2.077 (1.077, 4.004) |
| Pneumonia | 22 (2.016%) | <10 (0.917%) | 0.0326 | 2.2 (1.047, 4.624) |

**Supplemental Table S6**. 1day-30day analysis between Cases and controls.
